# Progranulin deficiency suppresses allergic asthma and enhances efferocytosis via PPAR‐γ/MFG‐E8 regulation in macrophages

**DOI:** 10.1002/iid3.779

**Published:** 2023-02-09

**Authors:** Qi Huang, Danlin Weng, Shifei Yao, Hailan Shen, Song Gao, Yanyu Zhang, Wenjie Huang, Yan Wang, Hong Wang, Wenchun Xu

**Affiliations:** ^1^ Key Laboratory of Laboratory Medical Diagnostics Designated by the Ministry of Education, School of Laboratory Medicine Chongqing Medical University Chongqing People's Republic of China; ^2^ Department of laboratory medicine The first affiliated hospital of Chongqing medical university Chongqing People's Republic of China; ^3^ Department of Laboratory Medicine, School of Laboratory Medicine, Affiliated Hospital of Zunyi Medical University Zunyi Medical University Zunyi People's Republic of China

**Keywords:** allergic asthma, efferocytosis, milk fat globule‐epidermal growth factor 8, peroxisome proliferators activated receptors γ, progranulin

## Abstract

Efferocytosis can resolve airway inflammation and enhance airway tolerance in allergic asthma. While previous work has reported that progranulin (PGRN) regulated macrophage efferocytosis, but it is unclear whether PGRN‐mediated efferocytosis is associated with asthma. Here, we found that in an ovalbumin (OVA)‐induced allergic asthma model, the airway inflammation was suppressed and the apoptosis in lung tissues was ameliorated in PGRN‐deficient mice. In contrast, PGRN knockdown in human bronchial epithelial cells increased apoptosis in vitro. Furthermore, PGRN‐deficient macrophages had significantly stronger efferocytosis ability than wild type (WT) macrophages both in vitro and in vivo. PGRN‐deficient peritoneal macrophages (PMs) exhibited increased expression of genes associated with efferocytosis including milk fat globule‐epidermal growth factor 8 (MFG‐E8), peroxisome proliferator‐activated receptor gamma (PPAR‐γ) and sirtuin1 (SIRT1) and increased capacity to produce the anti‐inflammatory mediator interleukin (IL)‐10 during efferocytosis. GW9662, the inhibitor of PPAR‐γ, abolished increased efferocytosis and MFG‐E8 expression in PGRN‐deficient PMs suggesting that PGRN deficiency enhanced MFG‐E8‐mediated efferocytosis through PPAR‐γ. Correspondingly, efferocytosis genes were increased in the lungs of OVA‐induced PGRN‐deficient mice. GW9662 treatment reduced MFG‐E8 expression but did not significantly affect airway inflammation. Our results demonstrated that PGRN deficiency enhanced efferocytosis via the PPAR‐γ/MFG‐E8 pathway and this may be one of the reasons PGRN deficiency results in inhibition of airway inflammation in allergic asthma.

## Graphical abstract

we found that progranulin (PGRN) deficiency conferred resistance to ovalbumin‐induced allergic asthma in mice and facilitated macrophage efferocytosis via peroxisome proliferator‐activated receptor gamma/milk fat globule‐epidermal growth factor 8 pathway. These results provide new evidence that PGRN influences the progression of allergic asthma.

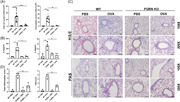

## 1 INTRODUCTION

Asthma is a chronic disease characterized by airway inflammation, airway hyperresponsiveness (AHR), and airway remodeling. It is typically treated with inhaled corticoids (ICS) alone or in combination with long‐acting beta‐agonists.^1^ However, long‐term ICS therapy can lead to adverse reactions and severe cases of refractory asthma can display glucocorticoid resistance.^2^ Despite the overall effectiveness of ICS treatment, the genetic basis for the disorder is currently unclear but this knowledge could enable development of novel protocols for treatment or prevention of asthma.

Under normal physiological circumstances, apoptotic cells are rapidly cleared by phagocytes in a process known as efferocytosis.^3^ These phagocytes express receptors and bridging molecules that specifically recognize phosphatidylserine (PS) exposed on dying cells. These receptors can be either membrane‐bound such as the CD36 scavenger receptor and T cell Ig and mucin domain (TIM) family proteins or circulating bridging molecules such as milk fat globule‐epidermal growth factor 8 (MFG‐E8) and growth arrest specific protein 6 (GAS6) and Protein S, the latter bridge dying cells and phagocytes via interactions with integrins and the TAM (Tyro3, AXL, and MerTK) receptor tyrosine kinases, respectively.^4^ The nuclear receptors peroxisome proliferators activated receptors (PPAR) and liver X receptors are also necessary components of efferocytosis.^5^, ^6^, ^7^ Efferocytosis differs from the phagocytosis of pathogens in that induces immunosuppression and anti‐inflammatory cytokines production.^4^ Due to the dual role of efferocytosis in inflammation resolution and immune tolerance, its effects on asthma are gaining increasing attention.^8^

The massive apoptotic cells, both structural and immune cells, seen in the airways of asthmatic patients is the result of long‐term stimulation by allergens, inflammatory cytokines, and oxidative stress.^9^, ^10^ The timely removal of apoptotic cells can prevent secondary necrosis and the release of proinflammatory mediators. Moreover, accelerating the apoptosis of eosinophils recruited and enhancing the clearance of apoptotic cells by phagocytes simultaneously were considered as novel approaches to asthma treatment.^11^ Evidence suggested that efferocytosis molecules have relevance to allergic inflammation states. Reduced expression of receptors such as Axl are also linked to decreased efferocytosis and to allergic inflammation states.^12^ MerTK deficiency impaired macrophage phagocytosis of apoptotic eosinophils leading to a delayed resolution of asthmatic inflammation.^13^ However, in addition to the physical removal of apoptotic cells from the lungs, efferocytosis enhances airway tolerance through modulation of macrophages production of anti‐inflammatory lipids and cytokines together with promotion of Treg cell generation. For example, defective efferocytosis attenuated prostaglandin E2 in severe asthma macrophages.^14^ Recently, apoptotic cell infusion has emerged as a therapeutic strategy for inflammatory lung diseases, including lung injury models (induced by LPS and bleomycin) and allergic asthma.^15^, ^16^, ^17^ Infusion of apoptotic cells resulted in increased efferocytosis by macrophages that displayed enhanced adenosine activity and also inhibited inflammatory cytokine production and promoted Treg activity. These all contributed to the relief of allergic airway inflammation.^15^ Therefore, it is possible to reduce airway inflammation by reversing weakened efferocytosis or by enhancing efferocytosis during asthma.

Progranulin (PGRN) is an autocrine growth factor that regulated numerous pathophysiological processes including tumorigenesis, neurodegeneration, wound healing, and embryonic development.^18^ PGRN also plays a role in immune diseases. Previous research suggested that PGRN bound to tumor necrosis factor receptors (TNFR) directly and block TNF‐α /TNFR signaling pathways, thus having a protective role in mice models of rheumatoid arthritis and inflammatory bowel disease.^19^, ^20^ In contrast, PGRN deficiency is protective against autoimmune encephalomyelitis in mice.^21^ However, there is some controversy as to whether PGRN has a protective or detrimental role in allergic asthma. A recent study demonstrates that PGRN aggravates allergic asthma via induction of type 2 cytokine production in NKT and airway epithelial cells.^22^ In contrast, exogenous PGRN alleviates airway inflammation and remodeling in chronic asthma models by suppressing high mobility group protein 1 expression and attenuates AHR by inhibiting of RAS homolog gene family member A (RhoA) activity.^23^, ^24^ Therefore, the definitive role of PGRN in asthma remains to be explored.

Interestingly, increased efferocytosis of PGRN‐deficient phagocytic cells may contribute to neuron loss and neurodegeneration^25^ and PGRN‐deficient mononuclear cells express high levels of CD36.^21^ These studies suggested that PGRN is involved in efferocytosis although a direct connection to asthma was not established. The current study examined whether PGRN can regulate efferocytosis and the role of PGRN‐mediated efferocytosis in asthma.

## 2 MATERIALS AND METHODS

### 2.1 Mice and groups

Wild C57BL/6 J mice were purchased from Tengxin. PGRN‐deficient mice (PGRN^‐/‐^, B6(Cg)‐Grntm1.1Aidi/J) were purchased from the Jackson Laboratory. All mice were reared and bred in the specific pathogen‐free laboratory of the Experimental Animal Center of Chongqing Medical University and housed at 22−24°C with a 12/12 h light/dark cycle and ad libitum access to food and water. All animal manipulations were under the strict guidance of the management regulations of the Experimental Animal Ethics Committee of Chongqing Medical University. For all experiments, age‐and sex‐matched wild type (WT) and PGRN‐deficient mice were used (6−8 weeks old). The mice were divided into five groups and the numbers of mice used for each experimental group are shown in the figure legends. The experimental groups were designed as follows: WT + phosphate‐buffered saline (PBS) group; WT + OVA group, wherein OVA was administered to induce allergic asthma inflammation; PGRN knockout (KO) + PBS group; PGRN KO + OVA group; PGRN KO + OVA + GW9662 group, wherein GW9662 was administered intraperitoneally (i.p.) 2 h before OVA challenge.

### 2.2 Antibodies and reagents

ELISA kits were obtained from BioLegend: IL‐4 (Cat. No. 431104) and IL‐5 (Cat. No. 431240). Antibodies used in our experiments were as follows: Anti‐cleaved caspase 3 (Cat. No 9664, clone 5A1E) and anti‐sirtuin 1 (SIRT1) (Cat. No. 3931 clone D60E1) were obtained from Cell Signaling Technology; anti‐MFG‐E8 (Cat. No. 377356, clone H‐3) was obtained from Santa Cruz; anti‐PPAR‐γ (Cat. No. ET1702‐57 clone JF101‐4) was obtained from HuaAn Biotechnology; anti‐PGRN (Cat. No. NBP1‐32076, polyclonal) was obtained from Novus Biologicals; anti‐GAPDH (Cat. No. 10494‐1‐AP polyclonal) was obtained from Proteintech and anti‐PE‐F4/80 Cat. No. 123109, clone BM8) was obtained from BioLegend. The following reagents were purchased from Sigma‐Aldrich; ovalbumin (OVA) (Cat. No. A5503‐50G); GW9662 (Cat. No. M6191); dexamethasone (Cat. No. D4902) and hydrogen peroxide (H_2_O_2_) (Cat. No. 323381). The following reagents were purchased from Thermo Fisher: CellTrace CFSE (Cat. No.C34570); lipofectamine 2000 (Cat. No. 11668019); Inject Alum (Cat. No. 77161). Recombinant M‐CSF (Cat. No. 315‐02) was from PeproTech. Iso plus (Cat. No. 9109) and PrimeScript RT reagent kit (Cat. No. RR037A) were purchased from Takara. siRNA was purchased from GenePharma. All primers and SYBR Green were purchased from Tsingke Biotechnology. The TUNEL kit (Cat. No. G1501) was obtained from Servicebio.

### 2.3 Induction of OVA‐induced airway inflammation

WT and PGRN‐deficient mice were used at 6−8 weeks of age and weighed 18−20 g. An allergic airway inflammation model was established as reported in our previous study (Wu et al.^26^ 2020). On Days 0 and 7 each mouse was injected i.p. with 100 μg OVA suspended with 1 mg aluminum hydroxide in 200 µL of sterile PBS. From Days 14 to 21, the mice were challenged with 5% OVA through nebulized inhalation every day for 30 min. The control group was sensitized and challenged with PBS in the same way. For the PGRN KO OVA + GW9662 group, PGRN‐deficient mice were given 1 mg/kg GW9662 i.p. 2 h before OVA challenge. On Day 22, mice were anesthetized with 1.5% sodium pentobarbital i.p. and killed.

### 2.4 Analysis of bronchoalveolar lavage fluid (BALF)

Tracheal intubation was performed using an indwelling needle after mice were anesthetized. BALF was obtained by 5 lavages using 1 mL pre‐cold PBS. Cell precipitates were obtained after centrifugation at 800*g* for 5 min at 4°C. The cell pellets were suspended in either 1 mL (WT OVA group) or 200 μL PBS (all other groups). Total numbers of inflammatory cells were counted using a modified Neubauer Counter under a microscope. Eosinophils were differentiated by Wright's staining.

### 2.5 Bone marrow derived macrophages (BMDM) isolation and culture

Bone marrow cells were collected from femurs of C57BL/6 J mice and differentiated into BMDM using medium supplemented with 10 ng/mL M‐CSF, 10% fetal bovine serum (FBS) and 1% penicillin/streptomycin (Gibco). Culture media was changed on Days 3 and 5 and cells were removed by scraping at Day 7 and centrifuged at 500*g* for 5 min to form a pellet and then resuspended in complete medium (without M‐CSF) for further use.

### 2.6 Peritoneal macrophages (PMs) isolation and culture

Sterilized liquid paraffin (1 mL) was injected i.p. into each mouse and at Day 5 the mice were euthanized by cervical dislocation. The peritoneal cavities were rinsed with 15 mL cold PBS and the cells were seeded in culture plates. After 30 min, nonadherent cells were removed with a PBS wash and recovered adherent PM were cultured in complete medium for further use.

### 2.7 Human bronchial epithelial (HBE) cell culture

The HBE cell line 16‐HBE was purchased from the American Type Culture Collection and cultured in 1640 medium supplemented with 10% FBS and 1% penicillin/streptomycin at 37°C with 5% of CO_2_. siRNA transfections were performed with Lipofectamine 2000. The sequences of siRNA are listed in Table 1.

**Table 1:** siRNAs used in this study.

	Sequences (5′‐3′)
siGRN‐homo‐427	GGCCACUCCUGCAUCUUUATT
siGRN‐homo‐557	UCCAAAGAUCAGGUAACAATT
siGRN‐homo‐598	CCUGAUAGUCAGUUCGAAUTT
siNC	UUCUCCGAACGUGUCACGUTT

### 2.8 RNA isolation and quantitative real‐time PCR

Total RNA was isolated from cells using Isoplus and reverse‐transcribed into cDNA using the PrimeScript reagent kit. Q‐PCR Primers were listed in Table 2. GAPDH was used as internal control to normalize relative gene expression using the $2^{‐\Delta \Delta C_{T}}$ method.

**Table 2:** Primers used in this study for PCR.

Gene	Forward	Reverse
mmu‐*Gapdh*	TGGCCTTCCGTGTTCCTAC	GAGTTGCTGTTGAAGTCGCA
mmu‐*Pparg*	CGGTTTCAGAAGTGCCTTG	GGTTCAGCTGGTCGATATCAC
mmu‐*Mfge8*	CCGCCTCGTCTGTGTATATGG	CTTGCTATCATAGTTGCTGGCT
mmu‐*Il10*	GCTGGACAACATACTGCTAACC	ATTTCCGATAAGGCTTGGCAA
homo‐*GRN*	GCCTTCTGCGACCTGGTTCAC	GACACATGACCGAGCTGGACAAG
homo‐*GAPDH*	TGCACCACCAACTGCTTAGC	GGCATGGACTGTGGTCATGA

### 2.9 Western blot

Cells and lung tissues were lysed using RIPA lysis buffer supplemented with 1% PMSF and proteins were separated using SDS‐PAGE and electrotransferred to PVDF membranes (Millipore, Cat. No. GVWP10050). The membranes were incubated with primary antibodies at 4°C overnight and washed with TBST and incubated with HRP‐conjugated secondary antibodies for 1 h at 37°C and protein bands were visualized using Image Lab.

### 2.10 Induction of apoptotic thymocytes

To induce apoptotic thymocytes, thymocytes from 4 to 6‐week‐old C57 mice were collected and cultured in RPMI‐1640 medium containing 10% FBS, 1% P/S, and 2.5 μM dexamethasone for 8 h. The rate of apoptosis was assessed using Annexin V/PI staining with flow cytometry.

### 2.11 Apoptotic cell phagocytosis assay in vitro and in vivo

BMDMs or PMs from WT and PGRN KO mice were plated on coverslips and incubated overnight and then cocultured with apoptotic thymocytes stained with CellTrace CFSE (1:1000 dilution) in 5:1 ratio of thymocytes: macrophages for 1 h. The cells were then fixed with 4% paraformaldehyde for 30 min then anti‐fade mounting medium was added and the images were viewed and collected by fluorescence microscope. In cases where apoptotic thymocytes were not stained, BMDMs were washed three times with pre‐cold PBS after completion of phagocytosis, then a coverslip with macrophages attached was stained with Wright's stain. The efferocytosis index was evaluated using microscopy and at least 500 macrophages per sample were evaluated. The relative in vitro efferocytosis index was calculated as the ratio of the number of BMDMs that phagocytosed apoptotic thymocytes relative to total BMDMs.

For some experiments, PMs were treated with the PPAR‐γ inhibitor GW9662 (50 mM) 24 h before adding CFSE‐labeled apoptotic thymocytes. For gene expression analysis of IL‐10, PMs were incubated with apoptotic thymocytes for 12 h. Thereafter, macrophages were washed three times with PBS before RNA isolation. Then the mRNA level of IL‐10 was analyzed by Q‐PCR.

WT and PGRN‐deficient mice were injected i.p. with 1 mL 3% thioglycolate and after 72 h each animal received 1 × 10^7^ CFSE‐labeled apoptotic thymocytes i.p and the mice were killed 1 h later. The efferocytosis index was determined using flow cytometry and phagocytic macrophages were identified as CFSE^+^F4/80^+^ cells.

### 2.12 Flow cytometry analysis

For phagocytosis assay in vivo, the isolated cell suspension was washed with PBS and were stained with PE anti‐mouse F4/80 (Cat. No. 123109, clone BM8). Antibody staining was performed at 4°C for 30 min. Measurements were performed at the BD FACSAria Ⅱ. Both PE and CFSE were used a 488 nm excitation laser, but PE was used a 585/42 bandpass filter while CFSE was used 530/30 bandpass filter. The gating was done using FlowJo Version 10. and the gating strategy was shown in the Figure 3E. For apoptosis analysis, cells were stained with the APC‐Annexin V Apoptosis Detection Kit with PI and analyzed using CytoFLEX flow cytometer (Beckman).

### 2.13 Histological analysis

The whole lung was fixed with 4% paraformaldehyde and embedded in paraffin. Lung tissue sections were stained with H&E and PAS and analyzed by light microscope (Nikon eclipse 80i).

### 2.14 TUNEL assay

Paraffin sections were deparaffinized and rehydrated and digested with proteinase K and permeabilized using the instructions of the manufacturer of the TUNEL kit (see above) and analyzed by fluorescent microscopy.

### 2.15 ELISA

The protein levels of IL‐4 and IL‐5 were detected by commercial ELISA kits according to the manufacturer's instructions (see above).

### 2.16 Statistical analysis

Data from at least two independent experiments were calculated with a statistical software package (GraphPad Prism 8.0). Data were presented as the means ± standard deviation (SD) and the group mean values were analyzed by the unpaired Student's *t* test or one‐way analysis of variance as appropriate. (Welch's *t* test was used when the variance of the two independent samples was not equal). *p* Values less than .05 were considered statistically significant, which is indicated by *p < .05; **p < .01; ***p < .001, ****p < .0001.

## 3 RESULTS

### 3.1 OVA‐induced allergic airway inflammation was suppressed in PGRN‐deficient mice

**Figure 1:**
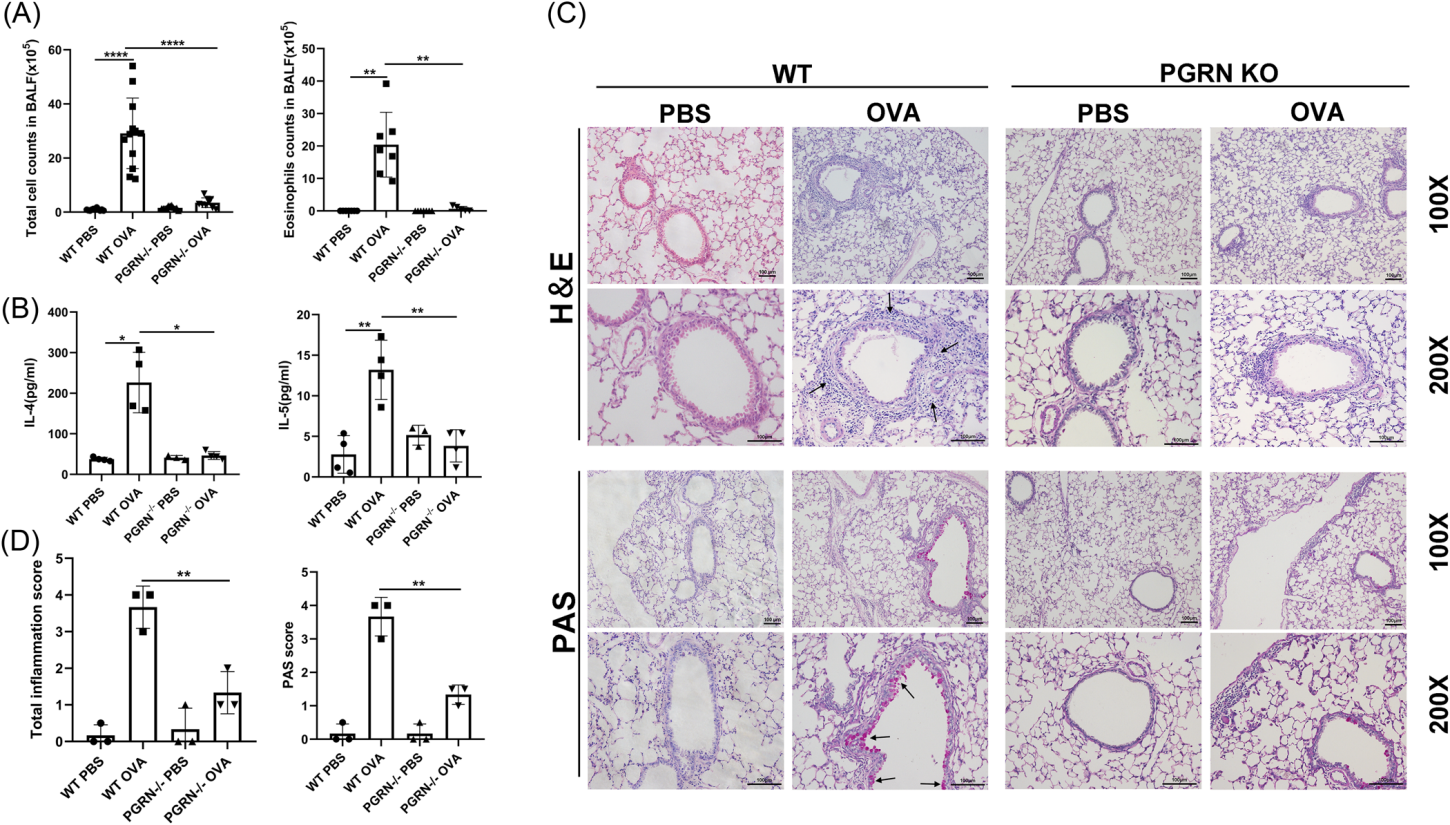
Ovalbumin induced allergic airway inflammation was inhibited in progranulin‐deficient mice. (A) Numbers of inflammatory cells in BALF of WT and PGRN KO mice induced (or not) by OVA. Results were shown as means ± SD. (WT PBS, *n* = 11, WT OVA, *n* = 12, PGRN KO PBS, *n* = 11, PGRN KO OVA, *n* = 9) **p < .01; ****p < .0001, Welch's *t*‐test. (B) Th2 cytokines IL‐4 and IL‐5 in lung homogenates measured by ELISA. Data were presented as means ± SD. (WT PBS, *n* = 4, WT OVA, *n* = 4, PGRN KO PBS, *n* = 3, PGRN KO OVA, *n* = 4). *p < .05; **p < .01, Welch's *t*‐test for IL‐4, unpaired *t* test for IL‐5. (C) Representative images of H&E and PAS staining lung tissue showing pathological alterations (arrowheads) and semi‐quantified score (D). Scale bar: 100 μm. The study was repeated for three times. BALF, bronchoalveolar lavage fluid; H&E, hematoxylin and eosin; KO, knockout; PAS, Periodic Acid‐Schiff; PBS, phosphate‐buffered saline.

To determine the role of PGRN in allergic asthma, we first established an OVA‐induced asthma model using WT and PGRN KO mice, and then collected BALF and lung tissue to evaluate airway inflammation. We found that OVA‐induced PGRN‐deficient mice possessed significantly lower numbers of inflammatory cells and lower Th2 cytokine production compared with OVA‐induced WT mice (Figures 1A,B). Histological examination of the lungs indicated peritracheal inflammatory cell infiltration and mucus production in OVA‐induced PGRN‐deficient mice was also significantly reduced compared with OVA‐induced WT mice (Figure 1C). These results suggested that knockout of PGRN can alleviate OVA‐induced allergic asthma in mice.

### 3.2 Deletion of PGRN suppressed apoptosis in lung tissues exposed to OVA, but aggravated the epithelial cell apoptosis in vitro

**Figure 2:**
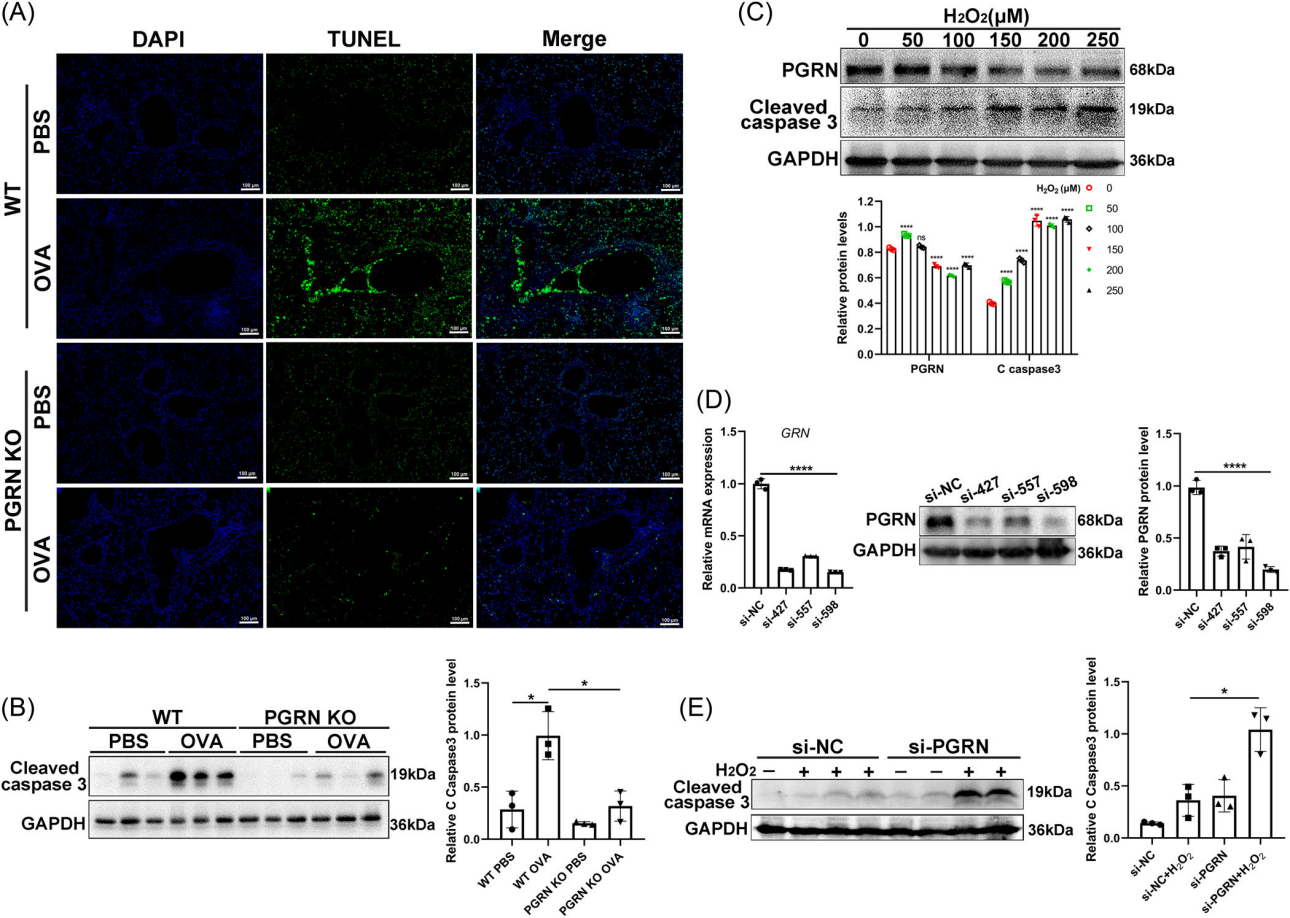
Deletion of progranulin suppressed apoptosis in the airways exposed to ovalbumin, but aggravated the human bronchial epithelial cells apoptosis in vitro. (A) Representative images of lung sections from WT and PGRN KO mice stained for TUNEL. Scale bar: 100 μm. (B) The protein levels of cleaved caspase 3 in mice lung were analyzed by Western blot. The results of densitometric analysis were showed as means ± SD (*n* = 3). *p < .05, unpaired t test. (C) Western blot analysis of PGRN and cleaved caspase 3 in 16‐HBE cells treated (or not) with H_2_O_2_ for 48 h. The results of densitometric analysis were showed as means ± SD (*n* = 3). ****p < .0001, one‐way ANOVA. (D) The knockdown efficiency of siRNA targeting PGRN was identified at the mRNA and protein levels 24 and 48 h following transfection, respectively. The relative mRNA expression and densitometric analysis were showed as means ± SD (n = 3). ****p < .0001, one‐way ANOVA. (E) Following knockdown of PGRN for 24 h, 16‐HBE cells were treated (or not) with H_2_O_2_ for 24 h. The densitometric analysis of cleaved caspase 3 was presented as means ± SD (*n* = 3). *p < .05, unpaired *t* test. The study was repeated for three times. ANOVA, analysis of variance; DAPI, 4′,6‐diamidino‐2‐phenylindole; HBE, human bronchial epithelial cells; TUNEL, terminal deoxynucleotidyl transferase dUTP nick end labeling.

In models of asthma, apoptotic cells accumulate in the lung tissue.^10^, ^27^ So TUNEL assays were performed on lung tissue from WT and PGRN‐deficient mice. The OVA‐induced PGRN KO mice had significantly fewer TUNEL‐positive cells in lung tissues than did WT mice (Figure 2A). In addition, cleaved caspase 3 levels were also significantly reduced in PGRN KO mice (Figure 2B). These results indicated that deletion of PGRN reduced lung apoptosis in this model of asthma.

Oxidative stress plays an important role in the pathogenesis of asthma^28^ and H_2_O_2_ is often used to induce apoptosis in airway epithelial cells that can mimic the oxidative stress seen in bronchial asthma.^29,30^ We used the HBE cells line 16‐HBE and exposed them to different levels of H_2_O_2_ for 48 h. We found that cleaved caspase 3 levels were increased while the expression of PGRN was decreased in a dose‐dependent manner (Figure 2C). This indicated that the H_2_O_2_‐induced apoptosis of 16‐HBE cells was negatively correlated with PGRN expression. We next used siRNA to knock down PGRN and siRNA‐598 showed the highest knockdown efficiency of both mRNA and protein (Figure 2D). 16‐HBE cells were treated with siRNA‐598 and H_2_O_2_ (250 μM) and PGRN knockdown directly led to the apoptosis of 16‐HBE and also significantly increased H_2_O_2_‐induced apoptosis (Figure 2E). These results indicated that PGRN knockdown aggravated human epithelial cell apoptosis in vitro.

### 3.3 PGRN deficiency in macrophages contributed to apoptotic cell clearance

Previous studies have demonstrated that loss of PGRN accelerates macrophage phagocytosis of apoptotic cells.^25^ Our results linked a PGRN knockdown with increased apoptosis using an in vitro model of oxidative stress but also apoptosis in the lungs of OVA‐induced PGRN KO mice was significantly lower than for WT mice. Therefore, we speculated that PGRN knockdown might enhance the clearance of apoptotic cells in asthmatic lung tissues.

**Figure 3:**
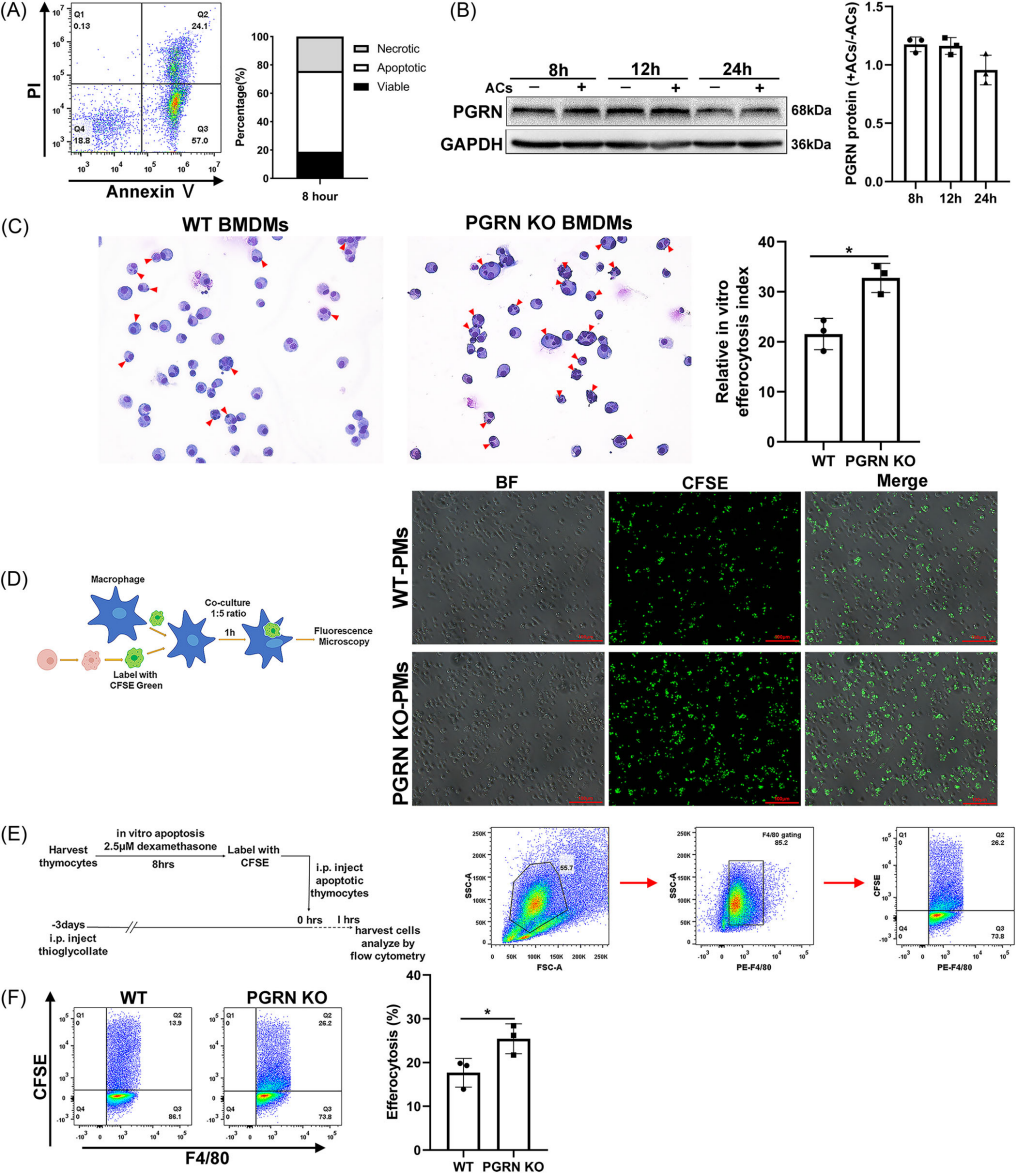
Progranulin deficiency in macrophages contributed to apoptotic cell clearance. (A) Assessment of apoptotic thymocytes by using Annexin V/PI staining with FCM. (B) WT PMs were co‐cultivated with apoptotic cells for 8, 12 and 24 h. PGRN was assayed by Western blot. (C) BMDMs were incubated for 1 h with a 5:1 ratio of ACs to BMDMs and assayed for efferocytosis by Wright's staining (X400). Relative efferocytosis index was calculated as the ratio of BMDMs with engulfed ACs (arrowheads) to BMDMs, at least 500 macrophages per sample were evaluated. Data were presented as means ± SD (n = 3). *p < .05, unpaired *t* test. (D) PMs were incubated for 1 h with a 5:1 ratio of CFSE‐labeled ACs to PMs and assayed for efferocytosis by image analysis of fluorescent microscopy images. Scale bar: 100 μm. (E−F) CFSE‐labeled apoptotic thymocytes were i.p. given to mice and “Efferocytosis (%)” is the percentage of peritoneal macrophages (F4/80^+^) containing CFSE‐related fluorescence was quantified by FCM. The gating strategies used for FCM (E). Data were shown as means ± SD (*n* = 3) (F). *p < .05, unpaired t test. The study was repeated for 3 times. ACs, apoptotic cells; BF, bright field; BMDMs, bone marrow derived macrophages; FCM, flow cytometry; PMs, peritoneal macrophages; SD, standard deviation.

We treated thymocytes with dexamethasone to induce apoptosis that was assessed using Annexin V/PI staining and flow cytometry (Figure 3A). To investigate variations in macrophage PGRN expression associated with efferocytosis, we added apoptotic thymocytes to PM from WT mice, and we found that PGRN expression did not change significantly after 8, 12, and 24 h of cocultivation (Figure 3B). Apoptotic thymocytes were then added to WT or PGRN KO BMDMs and efferocytosis was evaluated using Wright's staining after 1 h cocultivation. We found that PGRN‐deficient BMDMs had significantly stronger efferocytosis ability than WT BMDMs (Figure 3C). In addition, CFSE‐labeled apoptotic thymocytes were added to PMs. The results indicated that higher levels of efferocytosis for PGRN‐deficient PMs than their WT counterparts (Figure 3D). We therefore extended these observations to in vivo experiments and CFSE‐labeled apoptotic thymocytes were given i.p. given to mice. We found that apoptotic cell engulfment by F4/80^+^ PMs in PGRN KO mice that was significantly enhanced compared with WT mice (Figure 3E). Taken together, the lack of PGRN resulted in increased clearance of apoptotic cells by macrophages.

### 3.4 PGRN deficiency enhanced MFG‐E8‐mediated efferocytosis through PPAR‐γ in macrophages

**Figure 4:**
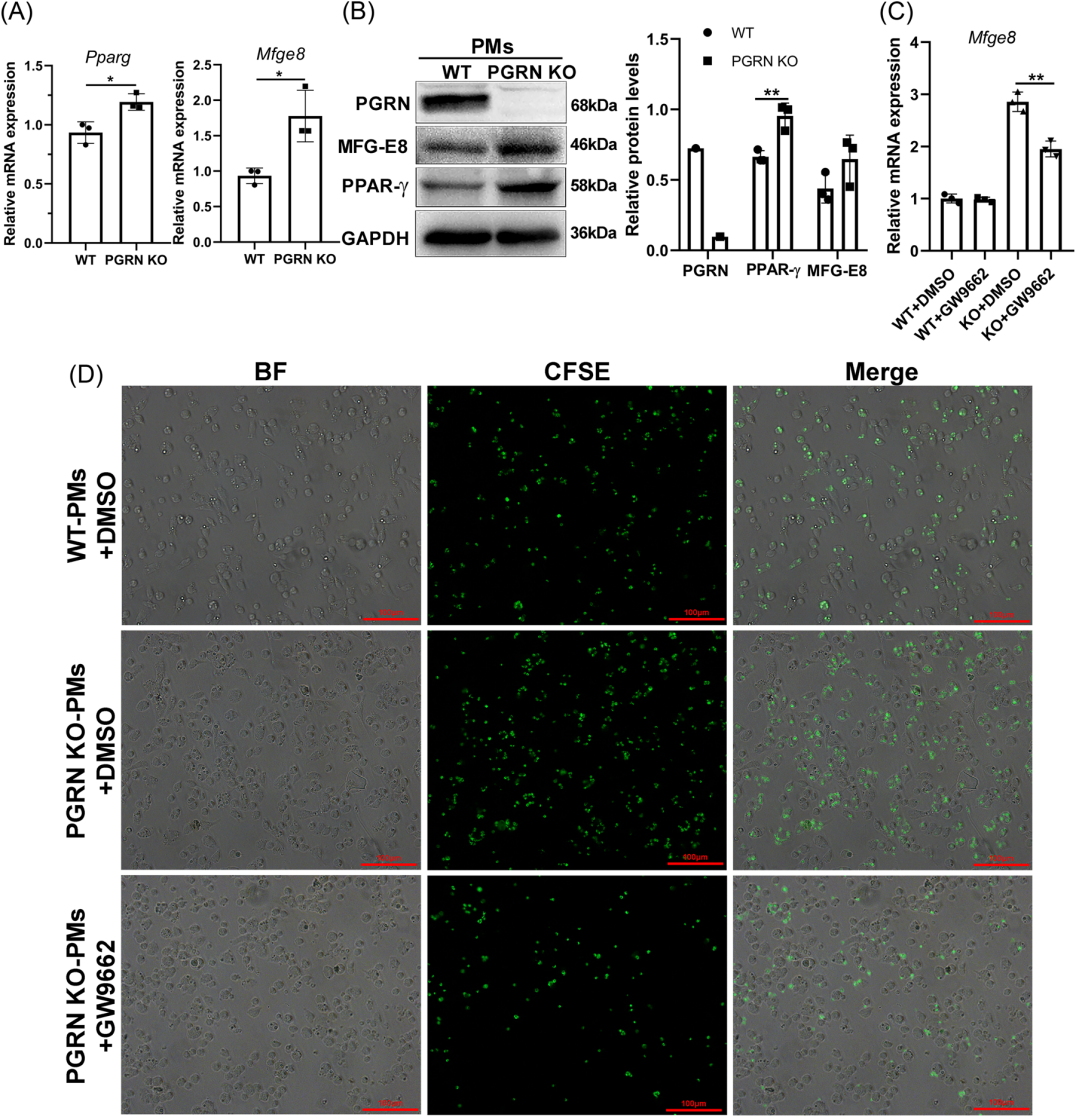
Progranulin deficiency in macrophages increased the expression of milk fat globule epidermal growth factor 8 through peroxisome proliferators activated receptors γ in macrophage. (A) mRNA was harvested from WT and PGRN KO macrophages and quantified by q‐PCR analysis for *Pparg* and *Mfge8*. The relative mRNA expression was showed as means ± SD (*n* = 3). *p < .05, unpaired *t* test. (B) Western blot analysis of PGRN, PPAR‐γ and MFG‐E8 in peritoneal macrophages. The results of densitometric analysis were showed as means ± SD (*n* = 3). **p < .01, unpaired *t* test. (C, D) Macrophages treated with DMSO or PPAR‐γ inhibitor GW9662 and assayed after 24 h for *Mfge8* mRNA (C) and efferocytic activity(D). Scale bar: 100 μm. The relative mRNA expression was showed as means ± SD (*n* = 3). **p < .01, unpaired *t* test. The study was repeated for 3 times. MFG‐E8, milk fat globule EGF factor 8; PPAR‐γ, peroxisome proliferators activated receptors gamma; SD, standard deviation.

MFG‐E8 is a secreted glycoprotein that bridges PS on apoptotic cells with integrin receptors on the surface of macrophages.^31^ The nuclear receptor PPAR‐γ has been shown to regulate the expression of engulfment receptors and bridging molecules such as MFG‐E8 to enhance efferocytosis.^32^, ^33^ We found the mRNA and protein levels of *Mfge8* and *Pparg* increased significantly in PGRN KO PMs (Figures 4A,B). To determine whether PGRN was regulating efferocytosis via PPAR‐γ, WT and PGRN KO PMs were treated with PPAR‐γ inhibitor GW9662. We found that *Mfge8* levels in PGRN KO macrophages were decreased following GW9662 treatment (Figure 4C) and consistent with these observations, GW9662 addition also impaired efferocytosis of PGRN KO macrophages (Figure 4D). These results indicated that PGRN deficiency in macrophages enhanced MFG‐E8‐mediated efferocytosis through the upregulation of PPAR‐γ.

### 3.5 PGRN deficiency led to increased SIRT1 expression through PPAR‐γ and promoted IL‐10 production in efferocytes

**Figure 5:**
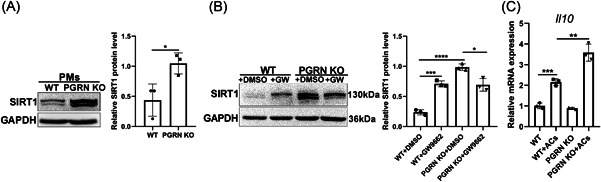
Progranulin deficiency led to increased Sirtuin1 expression through peroxisome proliferators activated receptors‐γ and promoted interleukin‐10 production in efferocytes. (A) Western blot analysis of SIRT1 in PMs. The results of densitometric analysis were showed as means ± SD (*n* = 3). *p < .05, unpaired *t* test. (B) PMs were treated with DMSO or PPAR‐γ inhibitor GW9662 and assayed for SIRT1 protein after 24 h. The results of densitometric analysis were showed as means ± SD (*n* = 3). *p < .05, ***p < .001, ****p < .0001, unpaired *t* test. (C) PMs were cocultured with apoptotic cells for 12 h and *Il10* levels were detected by q‐PCR. The relative mRNA expression was showed as means ± SD (n = 3). **p < .01, ***p < .001, unpaired *t* test. The study was repeated for three times. GW, GW9662; PM, peritoneal macrophage; PPAR‐γ, peroxisome proliferator‐activated receptor gamma; SD, standard deviation; SIRT1, sirtuin 1.

PPAR‐γ can also be an upstream negative regulator of NAD^+^‐dependent histone deacetylase SIRT1 by inhibiting the deacetylase activity and transcription.^34^ However, the activation of PPAR‐γ in macrophages exerts anti‐inflammatory effects by increasing SIRT1 levels.^35^ We found that the expression of both PPAR‐γ and SIRT1 were significantly increased in PGRN‐deficient macrophages compared with WT (Figure 5A). Subsequently, we explored whether the increased SIRT1 in PGRN KO macrophages was mediated by PPAR‐γ. We found SIRT1 expression increased following GW9662 treatment in WT PMs (Figure 5B). These results may be related to the negative feedback loop and self‐regulation loop.^34^ However, SIRT1 increased were also significantly inhibited by GW9662 in PGRN KO PMs (Figure 5B). Therefore, although the regulation of SIRT1 by PPAR‐γ differed between WT and PGRN KO macrophages, our data indicated that PGRN KO macrophages can increase SIRT1 expression via upregulating PPAR‐γ. SIRT1 protein levels are also elevated during efferocytosis and anti‐inflammatory cytokine IL‐10 expression in efferocytes is dependent on SIRT1.^36^ We found a similar baseline level (without apoptotic cell treatment) of IL‐10 in WT and PGRN KO PMs, but the expression of IL‐10 was significantly increased in PGRN KO compared with WT PMs after apoptotic cell treatment. (Figure 5C). These data suggested that PGRN deficiency promoted IL‐10 production in macrophages in an efferocytosis‐dependent manner and may be related to the high expression of SIRT1.

### 3.6 PGRN deficiency promoted the expression of MFG‐E8 by upregulating PPAR‐γ in lung of asthmatic mice

**Figure 6:**
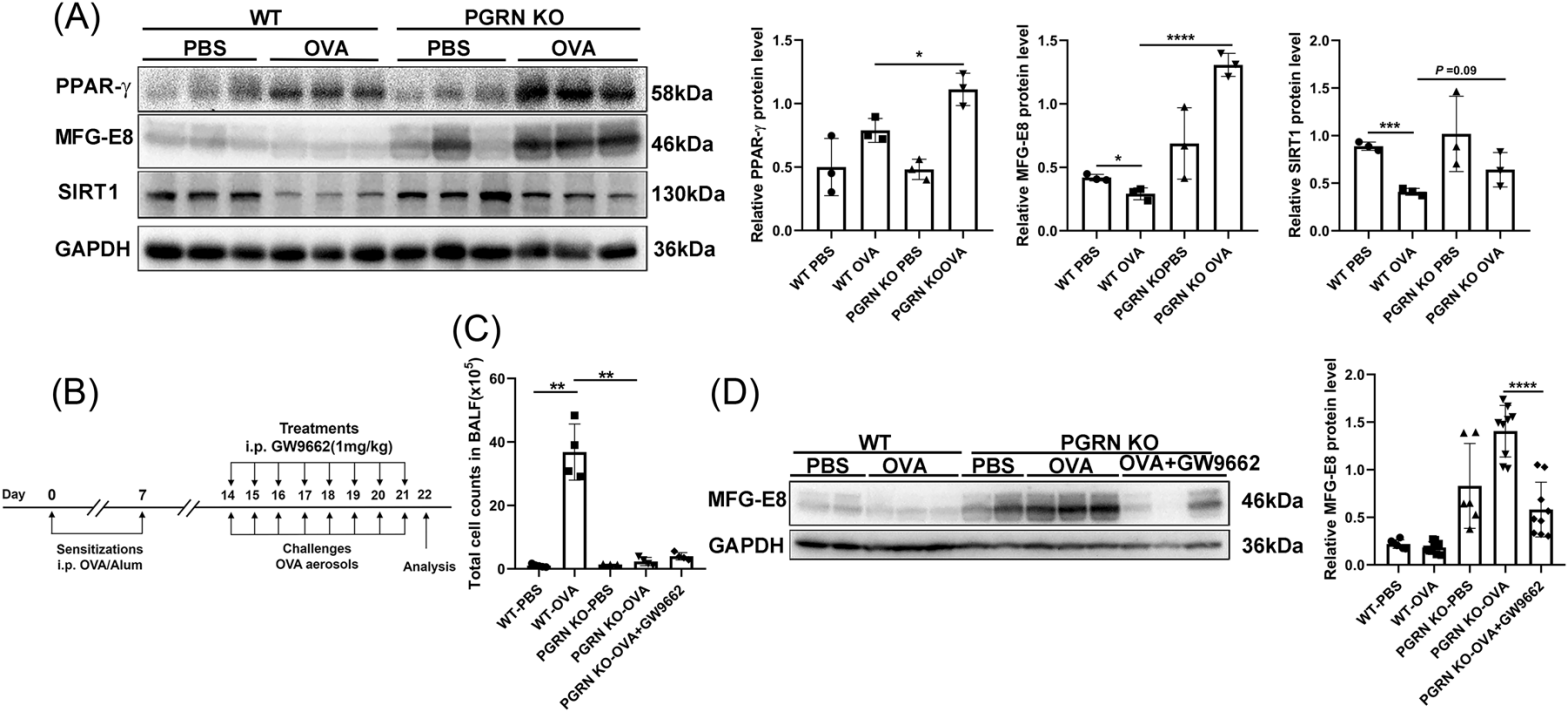
Progranulin deficiency promoted the expression of milk fat globule‐epidermal growth factor 8 by upregulating peroxisome proliferators activated receptors‐γ in lung of asthmatic mice. (A) Protein levels of PPAR‐γ, MFG‐E8 and SIRT1 in mice lungs were analyzed by Western blot. The results of densitometric analysis were showed as means ± SD (*n* = 3). *p < .05, ***p < .001, ****p < .0001, unpaired *t* test. (B) Protocol of establishing OVA‐induced allergic airway diseases model. (C) The total number of leukocytes in BALF. Results were shown as means ± SD. (WT PBS, *n* = 5, WT OVA, *n* = 4, PGRN KO PBS, *n* = 3, PGRN KO OVA, *n* = 4, PGRN KO OVA + GW9662, *n* = 4) **p < .01, Welch's *t*‐test. (D) Protein levels of MFG‐E8 in mice lungs was analyzed by Western blot. (*n* = 2 or 3). The study was repeated for three times. Alum, aluminum; OVA, ovalbumin; PGRN, Progranulin; SD, standard deviation; WT, wild type.

To further examine the effects of PGRN‐mediated macrophage efferocytosis on asthma, we measured expression levels of efferocytosis‐related molecules in OVA‐induced WT and PGRN KO mice. The levels of the key bridging molecule MFG‐E8 in the OVA‐induced PGRN‐deficient mice was significantly higher than that of OVA‐induced WT mice. Notably, PGRN KO mice also expressed higher MFG‐E8 compared with WT mice in the PBS groups (Figure 6A). These results indicated that PGRN deficiency may enhance the clearance of apoptotic cells in lung tissue consistent with the results of OVA‐induced apoptosis in the lungs of PGRN‐deficient mice. PPAR‐γ expression in OVA‐induced PGRN‐KO mice was significantly increased compared with the WT‐OVA group (Figure 6A), and the PPAR‐γ inhibitor GW9662 treatment decreased expression of MFG‐E8 in the lungs of OVA‐induced PGRN KO mice (Figure 6D). This suggested that PGRN deficiency promoted MFG‐E8 expression by upregulating PPAR‐γ in the lungs of asthmatic mice. We also found that PPAR‐γ inhibitor treatment resulted in an increased (but not significant) difference in the numbers of inflammatory cells in BALF of OVA‐induced PGRN KO mice (Figure 6C).

SIRT1 is expressed at low levels in the lungs of OVA‐induced asthmatic mice and the activation of SIRT1 can inhibit the onset of asthma.^37^, ^38^ We found that the expression of SIRT1 in OVA‐induced PGRN KO mice was higher than that in WT asthmatic mice suggesting an anti‐inflammatory effect of SIRT1 in PGRN KO mice (Figure 6A).

## 4 DISCUSSION

In this study, we found that OVA‐induced airway inflammation was significantly suppressed in PGRN‐deficient mice, consistent with a proinflammatory role but not with the anti‐inflammatory role of PGRN in allergic asthma.^22^, ^23^, ^24^ Therefore, in our study, we used PGRN deficient mice to investigate the role and possible mechanisms for PGRN regulation of the development of allergic inflammation.

We found that apoptotic cells in the lungs were significantly reduced in PGRN‐deficient mice compared with their WT counterparts. However, both our data and previous studies demonstrated that knockdown of growth factor PGRN in airway epithelial cells can trigger apoptosis in vitro.^39^ Generally, increased apoptosis of epithelial cells disrupts the barrier function that allows increased penetration of harmful substances such as allergens.^40^ Also, apoptotic cells, if not removed in time, lead to secondary necrosis, all of which could aggravate the allergic inflammatory response. Therefore, a reduction in apoptotic cells in the lungs of OVA‐induced PGRN‐deficient mice may be due to accelerated clearance of apoptotic cells.

PGRN‐deficient macrophages can phagocytize apoptotic cells to a greater degree compared with WT.^25^ In asthma, efferocytosis was thought not only to remove apoptotic cells from the lungs, but also to enhance airway tolerance through modulation of macrophages production of anti‐inflammatory lipids and cytokines, inhibiting inflammatory cytokine production together with promotion of Treg cell generation. Enhanced macrophage efferocytosis by apoptotic cell infusion is considered a promising therapeutic strategy to alleviate allergic airway inflammation.^15^ Indeed, our results indicated that PGRN KO BMDMs and PGRN KO PMs both have stronger ability than WT macrophages in vitro to perform efferocytosis and is consistent with previous reports.^21^, ^25^ PMs from PGRN‐deficient mice also possessed enhanced phagocytosis in vivo. Our data demonstrated that PGRN deficiency promoted IL‐10 production in macrophages in an efferocytosis‐dependent manner. These results support the idea that PGRN deficiency contributed to apoptotic cells removal and airways tolerance maintenance. Nevertheless, the reduction in apoptosis in the lungs, may be partially due to other immunomodulatory pathways being influenced in PGRN‐deficient mice that may contribute to the attenuation of the allergic inflammation phenotype.

Efferocytosis‐related molecules, such as the TAM receptor tyrosine kinase family, have been reported to be involved in the development of allergic airway inflammation. The shedding of AXL from the surface of airway macrophages in asthmatic patients is associated with impaired efferocytosis.^12^ In addition, MerTK deficiency impaired phagocytosis of apoptotic eosinophils by macrophages, leading to delayed resolution of asthmatic inflammation.^13^ Apoptotic cell lipids activate the nuclear receptor PPARs during efferocytosis resulting in an increase in expression of phagocytic receptors and bridging molecules such as CD36, Mertk, and MFG‐E8^33^, ^41^ and PGRN knock‐down in preadipocytes increased PPAR‐γ expression.^42^ In our study, PPAR‐γ and MFG‐E8 expression levels were significantly higher in PGRN KO compared with WT macrophages. In addition, an inhibitor of PPAR‐γ abolished the promotive effect of efferocytosis by reducing the transcription of MFG‐E8 partially. These results suggested that PGRN deficiency increased MFG‐E8 expression by upregulating PPAR‐γ and then promoted efferocytosis. PPAR‐γ activation in macrophages can also exert anti‐inflammatory effects through the increased expression of SIRT1.^35^ Our results demonstrated that PGRN KO macrophages increased SIRT1 expression via upregulating PPAR‐γ. Notably, SIRT1 is highly expressed in efferocytes and is required for IL‐10 production.^36^ The above evidence explains the increased expression of IL‐10 in PGRN KO efferocytes.

In the lung tissues of mice, we found that the protein levels of efferocytosis‐related molecules including PPAR‐γ and MFG‐E8 in PGRN KO OVA group were also significantly higher than those in WT OVA group. MFG‐E8 has been shown to be reduced in an OVA‐induced asthma model and plays a role in suppressing inflammation and airway hyper‐responsiveness that are primarily the result of MFG‐E8 effects on RhoA and PTEN pathways in smooth muscle cells.^43^, ^44^ However, there are no studies that have reported that MFG‐E8 affects asthma by regulating efferocytosis. The MFG‐E8 protein levels can be reduced by intraperitoneal injection of PPAR‐γ inhibitor to OVA‐induced mice. In addition, the numbers of BALF inflammatory cells did not significantly change although there was an increasing trend after GW9662 treatment. This may be a reflection of MFG‐E8 regulation by other transcription factors such as PPAR‐δ.^5^ PGRN deficiency also leads to the upregulation of complement factor C1qA^45^ and PGRN‐deficient mononuclear cells express elevated CD36 levels.^21^ Both CD36 and C1qA are important molecules in the recognition of apoptotic cells by macrophages.^46^ Moreover, PGRN can also bind to Gas6 and inhibit the Gas6‐Tyro3 interactions.^47^ Therefore, a possible reason the PPAR‐γ inhibitor treatment did not significantly aggravate asthmatic inflammation in PGRN KO mice was that it did not completely weaken PGRN‐mediated efferocytosis of lung macrophages.

Airway epithelial cells are nonprofessional phagocytes and dexamethasone can enhance phagocytosis of apoptotic eosinophils in human alveolar epithelial cells.^48^ Rac‐1‐ or IGF‐1‐mediated epithelial cell efferocytosis also critically influences allergic airway inflammation in mice.^49^, ^50^ However, Miki et al. reported that airway epithelial cells display a feeble capacity to phagocytose apoptotic cells compared with macrophages in a model given apoptotic cells intratracheally.^15^ Therefore, the objective of our study was primarily to explore the role of PGRN‐mediated macrophage efferocytosis in asthma. In other studies of efferocytosis in lung disease, PMs or RAW264.7 cells were used to replace alveolar macrophages.^51^, ^52^ Therefore, we chose to use more accessible PMs or BMDMs. However, due to the possible differences in the phenotypes of diverse macrophages, a limitation of our study is that we did not assess differences in efferocytosis of alveolar macrophages from untreated or asthmatic WT and PGRN‐deficient mice.

## 5 CONCLUSION

we found that PGRN deficiency conferred resistance to OVA‐induced allergic asthma in mice and facilitated macrophage efferocytosis via PPAR‐γ/MFG‐E8 pathway. These results provide new evidence that PGRN influences the progression of allergic asthma.

## AUTHOR CONTRIBUTIONS

Wenchun Xu and Qi Huang designed the studies and wrote the paper; Qi Huang, Danlin Weng and Shifei Yao performed animal and cell experiments; Qi Huang, Yanyu Zhang, Wenjie Huang and Yan Wang analyzed data; Hailan Shen, Song Gao and Hong Wang provided advice on experiments. All authors read and approved of the final manuscript.

## Data Availability

The data that support the findings of this study are available from the corresponding author upon reasonable request.
